# Calcified chondroid mesenchymal neoplasm: a case report of perineal involvement and literature review

**DOI:** 10.3389/fonc.2026.1783265

**Published:** 2026-03-13

**Authors:** Chenxi Weng, Yuanli Zhong, Liqian Hu, Gangping Wang

**Affiliations:** 1 Department of Nursing, the Fourth Affiliated Hospital of School of Medicine, and International School of Medicine, International Institutes of Medicine, Zhejiang University, Yiwu, China; 2 Department of Pathology, The Fourth Affiliated Hospital of School of Medicine, and International School of Medicine, International Institutes of Medicine, Zhejiang University, Yiwu, China

**Keywords:** calcified chondroid mesenchymal neoplasm, CCMN, chondroid matrix, FN1, perineum

## Abstract

Calcified chondroid mesenchymal neoplasm (CCMN) is a recently characterized solid tumor of bone and soft tissue. Its histological features include the formation of cartilage or chondroid matrix, while molecular characteristics are marked by the presence of *FN1* gene rearrangement. This article presents a rare case of CCMN occurring in the perineum of a 59-year-old female. The tumor exhibits the following characteristics: grossly, it appears as a solid nodule; histologically, it demonstrates lobulated growth, with polygonal, oval, or spindle-shaped cells observed within the chondroid matrix, alongside a significant number of osteoclast-like giant cells and calcium phosphate-like deposits. Molecular testing confirmed the presence of an *FN1* (exon 36):*:FGFR2* (exon 2) gene fusion through RNA sequencing. Immunohistochemical staining did not provide substantial assistance in diagnosing CCMN. Although recurrence occurred post-surgical resection, it was not common. Currently, there are no reported cases of metastasis. Existing literature primarily identifies such tumors in the distal extremities and temporomandibular joint; however, the case reported herein is the first documented occurrence in the perineum of a female. This finding suggests that the spectrum of CCMN may extend beyond the traditionally recognized locations of the distal extremities and temporomandibular joint.

## Introduction

Since 2001, a category of tumors defined by the presence of a cartilaginous matrix—with or without varying degrees of calcification—has been documented in the literature (1). Prior to this formal recognition, these tumors were described using a heterogeneous set of terms, including chondroid tenosynovial giant cell tumor (TGCT), calcified tendon membrane fibroma, calcium pyrophosphate dihydrate (CPPD) deposition disease (tophaceous pseudogout), and chondroblastoma, etc. (2–6). These tumors exhibit certain overlaps in their cellular morphology and histological characteristics. They not only possess similar chondroid-like soft tissue histological features of chondroma, but also exhibit features similar to tenosynovial giant cell tumors or calcium phosphate salt-like deposits. This makes it quite challenging to precisely classify and diagnose these morphologically soft tissue tumors through histological analysis. Subsequently, numerous literature studies have found that these tumors are closely related to the translocation of the *FN1* gene. In 2021, Liu, Y.J., et al. proposed that tumors forming a cartilage or cartilage-like matrix with specific morphological characteristics and often containing a gene fusion of the *FN1* receptor tyrosine kinase be collectively referred to as Calcified Chondroid Mesenchymal Neoplasm (CCMN) (7).In 2023, Kallen, M.E., et al., and in 2024, Benard, C., et al., conducted studies involving 33 cases each and agreed on the use of the universal terminology of CCMN to replace the previous heterogeneous nomenclature (8, 9). According to earlier reports, such tumors primarily occur in the temporomandibular joint (TMJ) and the distal extremities. In this article, we present a rare case of a calcified chondroid mesenchymal neoplasm (CCMN) occurring in the perineum, which contains a *FN1* (exon 36)::*FGFR2* (exon 2) gene fusion. Furthermore, we conducted a literature review and systematically analyzed the epidemiological, histological, molecular characteristics, and prognosis of this tumor.

## Case presentation

A 59-year-old female presented with a mass in the perineal region accidentally found over 10 years ago; the mass was approximately the size of a peanut at that time, with no accompanying symptoms such as pain, skin redness, ulceration or purulent discharge, and no special treatment was received. Over the past 10 years, the mass has gradually increased in size, currently reaching about the size of an egg. Other symptoms remain basically the same as before. There is no obvious abnormality in her family history. Physical examination revealed that a mass about 4*4 cm could be felt in her perineum. The texture was firm, the boundary was clear, and there was no tenderness.

**Figure 1:**
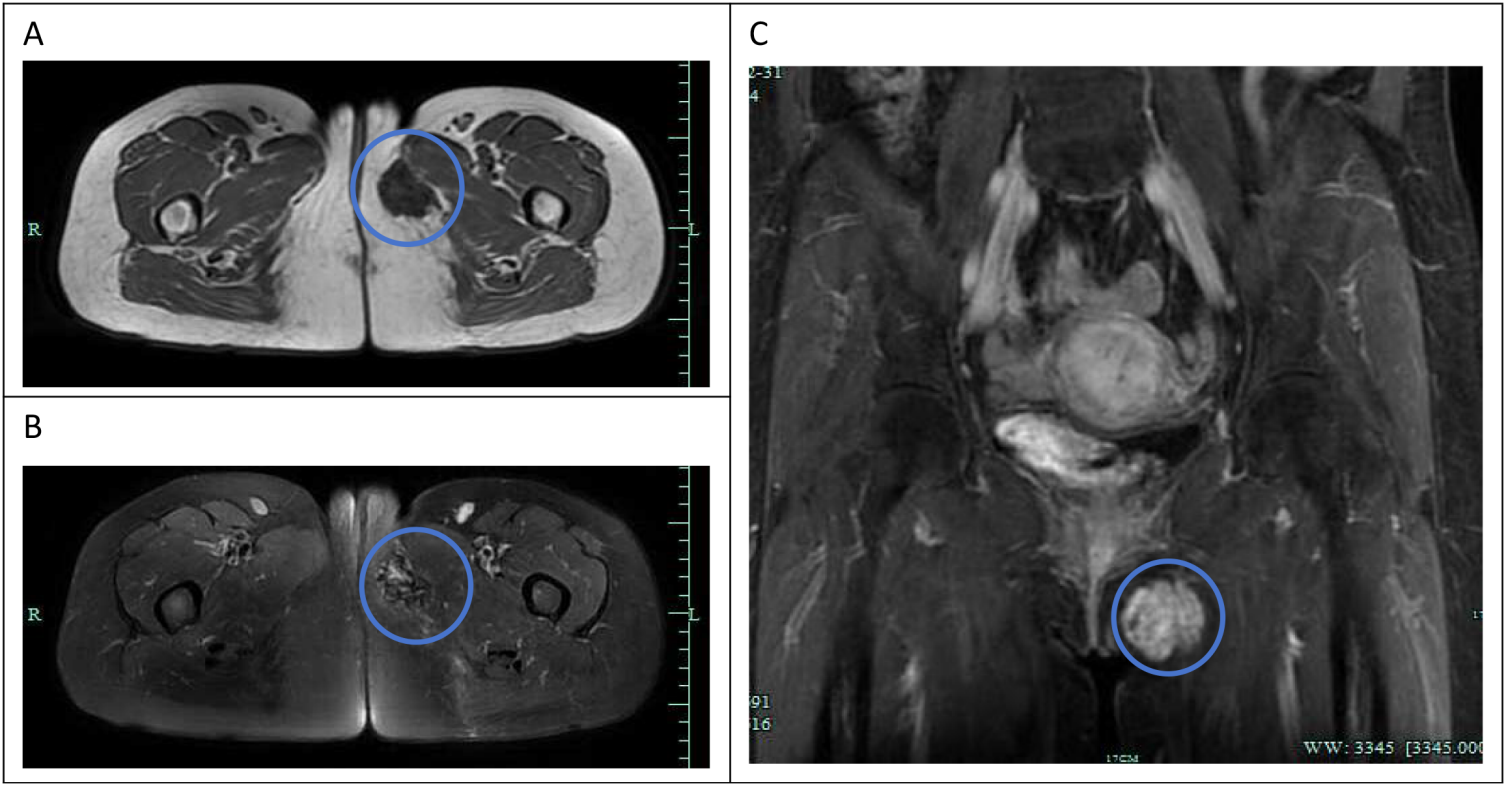
Pelvic MR enhanced 3.0T imaging: An irregular abnormal signal shadow is observed in the left perineum, approximately 35×27mm in size (red circle). **(A)** (transverse section) T1WI shows a slightly low signal. **(B)** (transverse section) T2WI shows a low signal. **(C)** (coronal section) after enhancement, it shows progressive enhancement and gradually fills towards the center.

Ultrasound examination revealed a heterogeneous echogenic mass measuring approximately 3.65 cm × 3.61 cm × 1.76 cm located in the subcutaneous tissue of the left perineal region. The mass exhibited relatively clear boundaries and a generally regular shape, with a small amount of blood flow signal detected both around and within the mass, resulting in a Resistive Index (RI) of 0.73. The ultrasound physician suggested that the mass is likely of soft tissue origin. An enhanced 3.0T pelvic MRI scan (**Figure 1**) demonstrated an irregular abnormal signal shadow in the left perineum, approximately 35 mm × 27 mm in size. On T1-weighted imaging (T1WI), it appeared as a slightly low signal (**Figure 1A**), while on T2-weighted imaging (T2WI), it presented as a low signal (**Figure 1B**). Following contrast enhancement, the mass exhibited progressive enhancement and gradually filled towards the center (**Figure 1C**). The radiologist initially suspected that this tumor might be a hemangioma.

**Figure 2:**
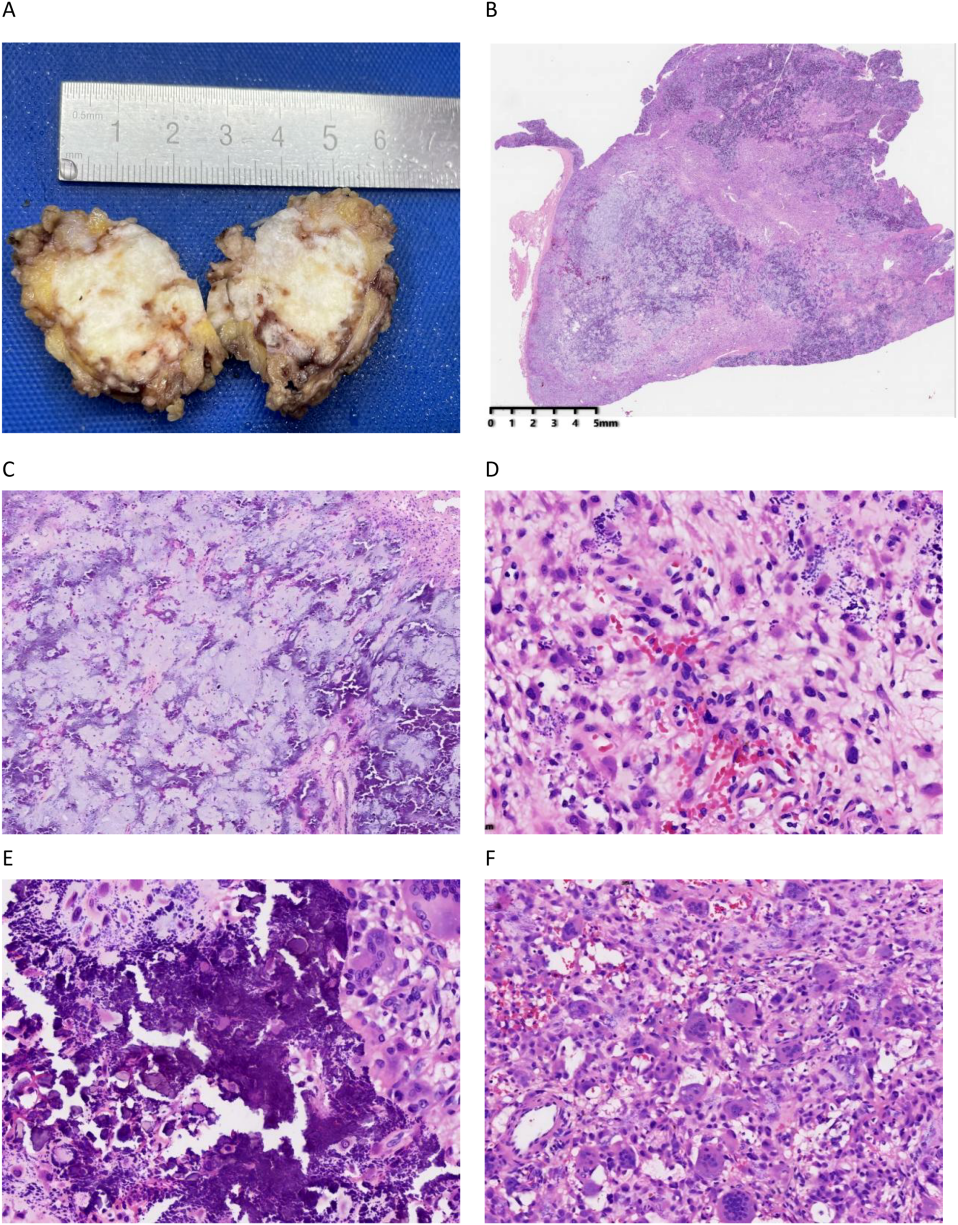
The gross and histological morphology of this case. **(A)** The gross cross-section of the tumor is nodular, measuring 3.3*2.7*2 cm. It is solid, grayish-yellow to grayish-white in color, with focal granular calcification. **(B)** The tumor exhibits a lobular growth pattern (hematoxylin and eosin [H&E], ×10). **(C)** The chondroid matrix and calcification are distributed in a disorganized manner (×40). **(D)** Within the chondroid matrix, polygonal, oval, spindle-shaped cells, and cells with eccentric nuclei are diffusely distributed, with eosinophilic cytoplasm (×100). **(E)** Calcification is accompanied by a surrounding foreign body reaction; crystalline deposits of calcium dihydrogen phosphate are visible (×200). **(F)** Abundant osteoclast-like giant cells are observed (×100).

The general surgeon performed a perineal tumor resection on the patient under combined intravenous-inhalation general anesthesia. During the operation, it was observed that the tumor had a clear boundary and was approximately 4*4 cm in size. After complete resection, it was sent to the pathology department for examination. Gross examination revealed that the section of the submitted tissue showed a nodular structure, measuring 3.3*2.7*2cm, being solid, grayish-yellow and grayish-white, with focal granular-like calcification (**Figure 2A**).

Histopathologically, the tumor growth presents a lobulated structure (**Figure 2B**), containing cartilaginous and collagenous matrices (**Figure 2C**), accompanied by extensive basophilic turbid calcification (**Figure 2D**). Polygonal, oval, and spindle-shaped cells are diffusely distributed throughout the tumor, with some nuclei displaced and the cytoplasm appearing eosinophilic (**Figure 2E**). Additionally, numerous giant cells resembling osteoclasts can be observed in certain areas (**Figure 2F**). The tumor shows no obvious pathological nuclear division images or necrosis. The resection margin of the mass was negative.

**Figure 3:**
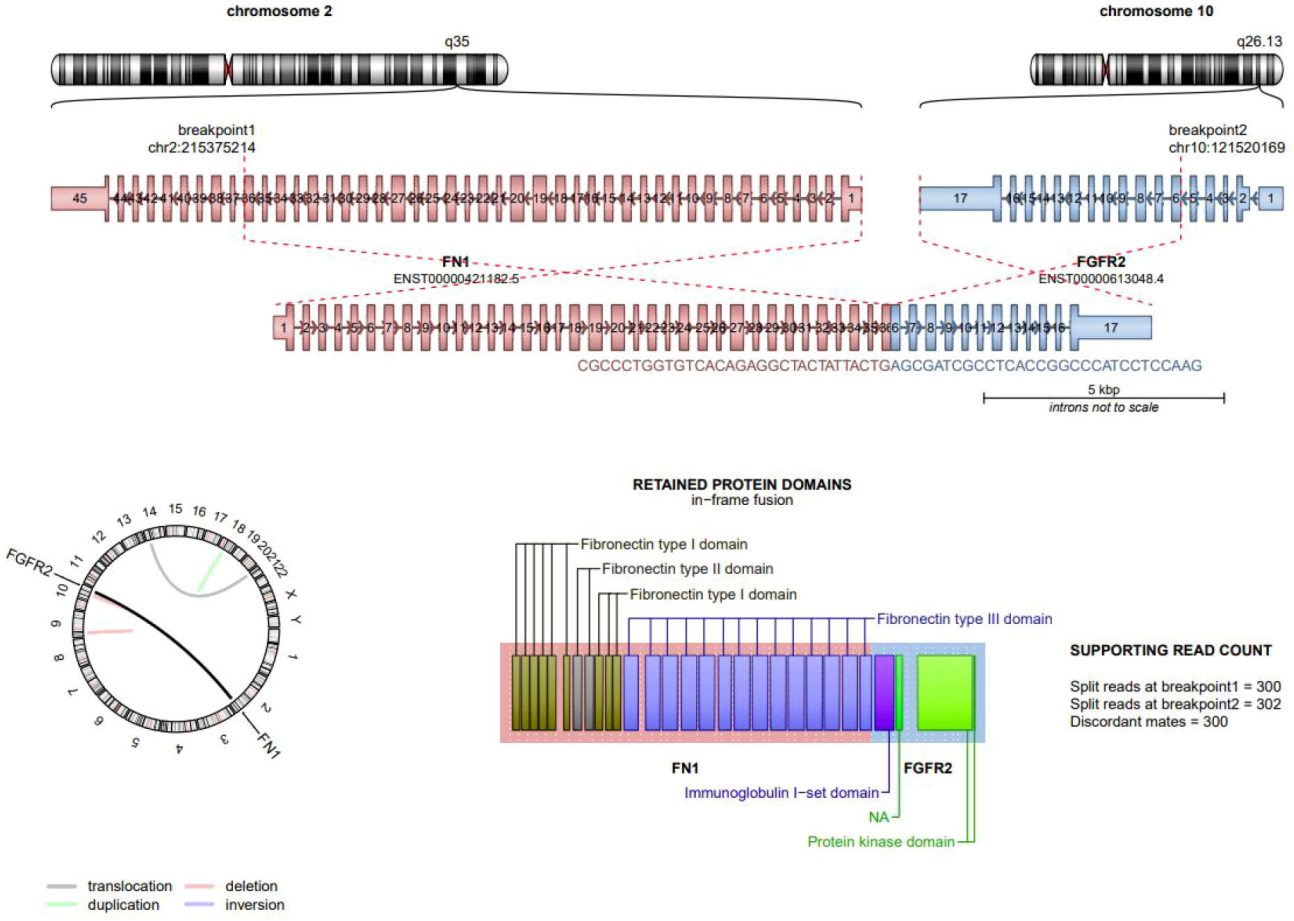
Next-generation sequencing (NGS) :*FN1-FGFR2* fusion sequence-structure characterization. The FN1 gene in the q35 region of chromosome 22 and the FGFR2 gene in the q26.13 region of chromosome 10 have fused. Gene and protein structure: A part of the exons of FN1 and a part of the exons of FGFR2 have fused. The fused protein retains the fibronectin type I, type II, and type III domains of FN1, as well as the immunoglobulin I-set domain and protein kinase domain of FGFR2.

Immunohistochemical stains indicated that the lesional cells were positive for ERG, Desmin, SSTR2, and NKX3.1. Additionally, giant cells expressed CD68. while, the immunohistochemical results for EMA, SMA, CD34, Myogenin, MyoD1, and Oligo2 were negative. Notably, INI-1 and Brg-1 were retained. Next-generation sequencing (NGS) identified an *FN1* (exon 36)::*FGFR2* (exon 2) fusion, which was subsequently confirmed by verification (**Figure 3**).

The overall morphological, immunohistochemical features, and characteristic molecular alterations (FN1-FGFR2 fusion) in this case are highly consistent with those of recently reported Calcified Chondroid Mesenchymal Neoplasm (CCMN). All of these findings support the diagnosis. At 1-year follow-up, no significant complications were observed, the patient recovered well, and there was no tumor recurrence.

## Discussion

Calcified chondroid mesenchymal neoplasm (CCMN) is a broad-term designation. First proposed as a new classification by Liu, Y.J., et al. in 2021, it encompasses a group of solid tumors found in bone and soft tissue, characterized histologically by the formation of a cartilaginous or chondroid matrix. These tumors share the molecular feature of *FN1* rearrangement, exhibit similar clinical biological behaviors, and demonstrate a high degree of overlap in histomorphology (7).

We searched and reviewed all relevant literature since the proposal of this new classification by Liu, Y.J., et al., and summarized the relevant clinicopathological features of calcified chondroid mesenchymal neoplasm (CCMN) (**Table 1**) (7–15).

**Table 1:** Summary of CCMN cases reported from 2021 onwards: clinicopathological features.

Author(s), year of publication	Case	Diagnosis	Sex	Age	Location	Size(cm)	Lobular	Chondroid matrix	Calcifications	Giant cells	Cytogeneticl/Molecular	Follow-up
Liu et al., 2021 (7)	12	Chondroid neoplasm of synovium	M	70	TMJ	4.00	Yes	Yes	Yes	Yes	FN1::FGFR2(E15::E7)	N/A
		Atypical chondroid neoplasm	F	66	TMJ	3.90	Yes	Yes	Yes	Yes	FN1::FGFR2(E42/E38:E5)	NED,12months
		TGCT	F	69	Foot	3.80	Yes	Yes	Yes	Yes	FN1::FGFR2(E31:E5)	NED,12 months
		TGCT	F	52	Hand	0.50	Yes	Yes	Yes	Yes	FN1::FGFR2(E11:E5)	N/A
		TPG	F	72	TMJ	N/A	Yes	Yes	Yes	Yes	FN1::FGFR2(E17::E5)	N/A
		TPG	M	44	TMJ	N/A	Yes	Yes	Yes	Yes	FGFRI::PLAGI(E1:E3)	N/A
		TGCT	M	36	Fifth finger-palm	N/A	Yes	Yes	Yes	Yes	FN1::FGFRI(E25::E9)	N/A
		Chondrocalcinosis(tophaceous pseudogout)	M	51	Thumb	N/A	Yes	Yes	Yes	Yes	FN1::MERTK (E24:E2)	N/A
		Giant cell-rich lesion with chondroid stroma	M	49	Temporal/external auditory canal	N/A	Yes	Yes	Yes	Yes	FN1::TEK(E27::E13)	N/A
		TGCT	F	22	TMJ/temporal	3.50	Yes	Yes	Yes	Yes	No fusion detected	NED
		TGCT	F	68	Index finger	2.00	Yes	Yes	Yes	Yes	FN1::NTRKI(E21:E7)	N/A
		TPG	M	69	Finger	4.00	Yes	Yes	Yes	Yes	No fusion detected	N/A
Georgant-zoglou et al, 2023 (10)	1	CCMN	F	67	Right index finge	N/A	Yes	Yes	Yes	Yes	FN1::FGFR3(E31::E3)	no significant change in the size ,3months
Christopher Warburton et al, 2023 (11)	1	CCMN	F	73	Right hamstring tendon	6.90	Yes	Yes	Yes	N/A	N/A	N/A
Kallen et al, 2023 (8)	33	N/A	M	41	Right index finger MP joint	2.10	N/A	Yes	N/A	N/A	N/A	NED,30months
		Osteo-cartilaginous proliferation”	M	67	Right index finger MP joint	0.80	N/A	Yes	N/A	N/A	N/A	NED,2months
		Soft tissue chondrom	M	26	Right foot,third digit	1.90	N/A	Yes	N/A	N/A	N/A	NED,24months
		Concerning cellular spindle cell tumor with cartilaginous features”	M	50	Left ring finger	1.50	N/A	Yes	N/A	N/A	No fusion detected	NED,23months
		Fracture vs. osteomyelitis vs. chondroid neoplasm	M	14	Toe	1.00	N/A	Yes	N/A	N/A	N/A	NED,21 months
		Calcifying aponeurotic fibroma	F	56	Right plantar foot	2.00	N/A	Yes	N/A	N/A	N/A	N/A
		Favor soft tissue chondroma	M	49	Left foot	3.50	N/A	Yes	N/A	N/A	N/A	NED,21months
		N/A	M	51	Right index finger	2.20	N/A	N/A	N/A	N/A	N/A	N/A
		N/A	F	72	Left hand	1.50	N/A	Yes	N/A	N/A	No fusion detected	N/A
		N/A	M	31	(multifocal)Right proximal forearm and right middle finger	2.9/2.4	N/A	Yes	N/A	N/A	No fusion detected	NED, 18 months
		“spindle cell neoplasm with giant cells, clinically recurrent	M	55	Left wrist	3.00	N/A	Yes	N/A	N/A	N/A	YES
		“Cartilaginous lesion”	F	87	Left index finger	2.30	N/A	Yes	N/A	N/A	N/A	NED,18months
		“Rule out malignancy”	F	36	Right foot tumor	1.30	N/A	Yes	N/A	N/A	N/A	N/A
		“Neoplasm with spindle cell, myxoid, and chondroid features”	F	45	Right index finger	4.00	N/A	Yes	N/A	N/A	N/A	NED,15months
		N/A	F	70	Right foot	5.20	N/A	Yes	N/A	N/A	N/A	N/A
		Giant cell tumor of tendon sheath with metaplastic cartilage	M	36	Right foot	1.30	N/A	Yes	N/A	N/A	N/A	N/A
		Chondroblastoma versus low-grade sarcoma	F	29	Left foot	2.00	N/A	N/A	N/A	N/A	N/A	N/A
		Bizarre parosteal osteochondromatous proliferation (BPOP	M	66	Right wrist	2.50	N/A	Yes	N/A	N/A	N/A	N/A
		“Epithelioid tumor with chondromyxoid stroma” possible glomus tumor, solitary fibrous tumor, fibroma of the tendon sheath, soft tissue chondroma, or myopericytom)	M	31	Left thumb	1.10	N/A	Yes	N/A	N/A	N/A	NED,6months
		sarcoma”; extraskeletal myxoid chondrosarcoma vs. chondro-blastic osteo-sarcoma	F	31	Left foot second digit	2.40	N/A	Yes	N/A	N/A	N/A	NED,6months
		ossifying fibromyxoid tumo	F	35	Left thumb cyst	1.10	N/A	Yes	N/A	N/A	N/A	N/A
		Osteo-cartilaginous tissue with calcifications	M	61	Right little finger	1.20	N/A	Yes	N/A	N/A	N/A	N/A
		Benign cartilaginous tumor	M	62	Right great toe	4.30	N/A	Yes	N/A	N/A	N/A	NED,6 months
		“Epithelioid proliferation and associated giant cells”	F	49	Right hand	2.00	N/A	Yes	N/A	N/A	N/A	NED,3months
		ow-grade chondrosarcom	M	48	Palmar aponeurosis	1.20	N/A	N/A	N/A	N/A	FN1:FGFR2	N/A
		Unusual benign chondroid lesions, most consistent with soft tissue chondroma	F	61	Hand/Phalan	1.90	N/A	N/A	N/A	N/A	N/A	N/A
		Phosphaturic mesenchymal tumor	F	61	H&N,Parotid	1.30	N/A	N/A	N/A	N/A	PLAGl and HMGA2 FISH negative	N/A
		Atypical chondroma with features of phosphaturic mesenchymal tumor	M	66	Subcutis of a finger/ distal phalanx	1.10	N/A	N/A	N/A	N/A	N/A	N/A
		Chondroma with extensive calcifications	F	65	Thumb-proximal phalanx	3.00	N/A	N/A	N/A	N/A	N/A	N/A
		Phosphaturic mesenchymal tumor	F	67	Finger	1.10	N/A	N/A	N/A	N/A	N/A	N/A
		N/A	M	69	Left fifth finger	1.50	N/A	Yes	N/A	N/A	FN1 rearranged	N/A
		N/A	F	36	TMJ	4.20	N/A	Yes	N/A	N/A	FN1 rearranged	N/A
		N/A	F	71	Right 3 digit plantar foot mass	2.40	N/A	Yes	N/A	N/A	Molecular testing failed due to insufficient RNA	NED,5months
Isidro Machado et al, 2024 (12)	1	CCMN	F	28	subungual lesion on her left hallux	N/A	Yes	Yes	Yes	Yes	FN1::FGFR2	NA
Chi et al, 2024 (13)	1	CCMN	F	41	Right TMJ region (including infratemporal fossa, masticator space, superficial parotid area)	4.90	N/A	Yes	Yes	Yes	N1::FGFR2 (E20::E7)	NED,7 months
Fisher et al, 2024 (14)	4	CCMN	F	36	Left second digit (subcutis)	3.10	Yes	Yes	Yes	Yes	PDGFRA (exon 22)::USP8 (exon 5) gene fusion	No
		CCMN	F	39	Left hip (capsule)	3.70	Yes	Yes	Yes	Yes	PDGFRA (exon 22)::USP8 (exon 5) gene fusion	No
		CCMN	F	61	Left middle finger	5.00	Yes	Yes	Yes	Yes	PDGFRA (exon 22)::USP8 (exon 5) gene fusion	N/A
		CCMN	F	62	Dorsal wrist	4.70	Yes	Yes	Yes	Yes	PDGFRA (exon 22)::USP8 (exon 5) gene fusion	Yes ,6 years
Clément Benard et al, 2024 (9)	33	CD	F	51	Foot	N/A	N/A	Yes	No	Yes	FN1::FGFR2(E39::E7)	N/A
		CD	F	62	Foot	N/A	N/A	Yes	Yes	No	FN1::FGFR2(E23::3/4/5)	N/A
		CD	F	72	TMJ	N/A	N/A	Yes	No	No	FN1::FGFR2(E20::E3)	N/A
		CD	M	32	Foot	N/A	N/A	Yes	Yes	No	No fusion	N/A
		CD	F	77	TMJ	1.50	N/A	Yes	Yes	Yes	FGFRI::FN1(NA)	N/A
		CD	M	13	Finger	N/A	N/A	Yes	Yes	No	COLIA2::MIR29BI(EI1::E4)	N/A
		CD	M	41	Foot	3.00	N/A	Yes	Yes	No	FN1:FGFR2(E27:E5)	N/A
		CD	M	26	Finger	2.30	N/A	Yes	No	No	No fusion	N/A
		CD	M	44	Temporal	N/A	N/A	Yes	Yes	Yes	FN1::AGFGI(E2:E2)	N/A
		CD	F	37	Foot	N/A	N/A	Yes	Yes	Yes	FN::FGFR2(E2::E10)	N/A
		CD	M	78	Foot	N/A	N/A	Yes	No	No	FN1::BMPR2(E19/46::E4/E13)	N/A
		CD	F	41	Foot	N/A	N/A	Yes	Yes	No	FN::PRG4(E46::E4)	N/A
		CD	F	11	Foot	N/A	N/A	Yes	Yes	No	FN1::FGFR2(E23.:E3)	N/A
		CD	NA	NA	Foot	N/A	N/A	Yes	Yes	No	FN1:FGFRI(E35::E9)	N/A
		TPG	M	64	Finger	5.50	N/A	Yes	Yes	No	FN1::FGFR2(E15::E3)	N/A
		TPG	F	85	Finger	N/A	N/A	Yes	Yes	No	FN1:FGFR2(NA)	N/A
		TPG	M	76	Finger	N/A	N/A	Yes	Yes	Yes	FN1::FGFR2(E26::E5)	N/A
		TPG	F	83	Foot	N/A	N/A	Yes	Yes	No	FN::FGFR2(E26::E7)	N/A
		TPG	F	82	Foot	N/A	N/A	Yes	Yes	No	FN1::FGFR2(E42::E6)	N/A
		TPG	F	63	Finger	N/A	N/A	Yes	Yes	Yes	FN::FGFR2(E20::E5)	N/A
		TPG	F	63	Hip	N/A	N/A	Yes	Yes	Yes	PDGFRA::USP8(E22::E5)	N/A
		TPG	F	58	TMJ	N/A	N/A	Yes	Yes	No	FN::FGFR2(E20::E3)	N/A
		TPG	M	56	TMJ	N/A	N/A	Yes	No	Yes	FN1::FGFR2(E2::E10)	N/A
		TGCT	F	33	TMJ	6.00	N/A	Yes	Yes	Yes	FN1::TEK(E27::E11)	N/A
		TGCT	M	51	TMJ	N/A	N/A	Yes	Yes	Yes	FN1::TEK(E24::E13)	N/A
		TGCT	M	47	Foot	2.30	N/A	Yes	Yes	Yes	FN1::FGFRI(E20::E5)	N/A
		TGCT	F	63	Foot	1.30	N/A	Yes	No	Yes	No fusion	N/A
		TGCT	F	60	Foot	2.50	N/A	Yes	Yes	Yes	PDGFRA::USP8(E22::E5)	N/A
		TGCT	F	55	TMJ	2.70	N/A	Yes	No	Yes	FN1::TEK(E27::EI2)	N/A
		TGCT	F	72	Temporal	N/A	N/A	Yes	Yes	Yes	FN1::TEK(E26::E12)	N/A
		TGCT	NA	46	Foot	1.50	N/A	Yes	Yes	Yes	FN1::FGFRI(E20::E3/4)	N/A
		TGCT	M	59	Hip	3.00	N/A	Yes	Yes	Yes	PDGFRA::USP8(E22::E5)	N/A
		TGCT	F	62	TMJ	N/A	N/A	Yes	Yes	Yes	PDGFRA::USP8(E22::E5)	N/A
IOANNIS PANAGOPOULOS et al, 2024 (15)	1	CCMN	F	71	Gastrocnemius muscle, subfascial	N/A	Yes	Yes	Yes	N/A	PDGFRA::USP8	N/A
Current case	1	CCMN	F	59	Perineum	3.30	Yes	Yes	Yes	Yes	FN1::FGFR2(E36::E2)	NED,12months

CCMN, calcified chondroid mesenchymal neoplasm; TGCT, tenosynovial giant cell tumor ; PMT, phosphaturic mesenchymal tumor; CD, soft tissue chondroma; TPG, tophaceous pseudogout; TMJ, temporomandibular joint; N/A, not available or not stated or N/A ; NED, no evidence of disease.

Among the 88 reported cases, including the present case, patients’ ages ranged from 11 to 87 years, with a mean age of 53 years. Tumor sizes varied from 0.5 to 6.9 cm, with an average of 2.68 cm. The tumors occurred in both males and females; however, there was a predominance of female patients over male patients (with a ratio of 51:35). Lesions primarily occurred in the distal extremities and the temporomandibular joint. The most frequently involved sites were the distal extremities (including the feet, hands, wrists, and forearms, n = 64) and the temporomandibular joint/temporal region/parotid gland region (n = 19). Additionally, lesions were rarely found in the soft tissues of the buttocks (n = 3), hamstring tendons (n = 1), and perineum (n = 1).

Grossly, these tumors present as solid, nodular masses. On cut surface, they appear grayish-white to grayish-brown, with a firm texture. In cases with significant calcification, the cut surface may have a gritty or sandy consistency on gross examination.

In tumors with available data, low-power microscopic observation revealed that all tumors (80/80) exhibited a lobular growth pattern. Tumor cells proliferated within abundant chondroid or cartilaginous matrix (21/21). Various forms of calcification were relatively common (48/55, 87%), with calcification morphologies ranging from rough, dirty, and lace-like to basophilic crystalline forms containing calcium phosphate crystal components. Tumor cells showed diverse morphologies, appearing polygonal, oval, epithelioid, or spindle-shaped. Some cell nuclei were eccentrically located, with abundant cytoplasm that ranged in color from eosinophilic to pale. Binucleation was occasionally observed. Osteoclast-like giant cells were visible in most cases (38/53, 72%). Pathological mitotic figures were absent or extremely rare, and no obvious necrosis was observed.

To date, no immunohistochemical markers have been identified as significantly beneficial in diagnosing calcified chondroid mesenchymal neoplasm (CCMN).

In the 12 cases reported by Liu, Y.J. et al. in 2021, 9 out of 12 cases (9/12) harbored fusions between the FN1 gene and receptor tyrosine kinase (TK) genes, specifically including FGFR2, MERTK, TEK, and NTRK1 (7). Subsequently, a 2023 study by Georgantzoglou, N. et al. identified an additional fusion between FN1 and FGFR3 (10).

In calcified chondroid mesenchymal neoplasm (CCMN), the most prevalent fusion partners of the FN1 gene are those encoding members of the fibroblast growth factor receptor (FGFR) family, which includes four types: FGFR1, FGFR2, FGFR3, and FGFR4. These receptors are located on the cell membrane and are distinguished by their extracellular ligand-binding domain, transmembrane helix, and intracellular tyrosine kinase domain. The binding of fibroblast growth factors (FGFs) to FGFRs leads to FGFR dimerization, activation of the tyrosine kinase domain, and initiation of downstream intracellular signaling cascades, such as RAS-MAPK, PI3K-AKT, and STAT pathways (16). FGF/FGFR signaling is crucial for embryogenesis and tissue homeostasis, as it regulates various cellular processes, including lineage specification, differentiation, proliferation, apoptosis, and migration (16). Animal studies have validated the significance of FGF/FGFR interactions in chondrogenesis, osteogenesis, and limb bud development (17).

In a 2024 report by Fisher et al., a novel PDGFRA::USP8 gene fusion was identified in four cases of calcified chondroid mesenchymal neoplasm (14).PDGFRα encodes platelet-derived growth factor receptor α (*PDGFRA*), a tyrosine kinase receptor that mediates cell proliferation, migration, and survival. *USP8* encodes a ubiquitin-specific protease involved in the downregulation of receptor tyrosine kinases, such as EGFR. The authors attempted to demonstrate the expression of PDGFRα and EGFR via immunohistochemistry; however, EGFR staining was negative, which ostensibly rules out this potential activation mechanism. The PDGFRA::USP8 fusion gene may be linked to cell proliferation, migration, and survival. This study enhances the genetic diversity of these tumors. Their findings suggest that FN1 fusion may not be a defining event for these tumors; rather, these tumors seem to share a unifying pathogenetic mechanism through protein kinase activation.

We summarized these studies (**Table 1**). Excluding 26 cases with unavailable data and 1 case with failed RNA extraction, 41 out of 61 cases exhibited *FN1* rearrangements, among which 24 cases involved the *FN1::FGFR2* fusion. Molecular data confirmed that FN1 fusion has the highest prevalence in CCMN, with *FGFR1* and *FGFR2* being the most common fusion partners. Additionally, the *PDGFRA::USP8* fusion appears to primarily affect large joints.

In terms of differential diagnosis, calcific tendinitis (HADD) should be considered first. HADD is a common cause of intratendinous mineralization, which predominantly affects females aged 30–50 years and mainly involves the shoulder joint. The underlying pathophysiological mechanism of hydroxyapatite deposition in the tendon has not been clearly defined, but it may be associated with repeated microtrauma. Such repeated microtrauma can lead to tendon ischemia, which in turn triggers tendon remodeling—with corresponding manifestations on imaging (18).On X-ray and CT, calcific tendinitis (HADD) may show amorphous calcifications and cortical erosion similar to those of CCMN. However, on contrast-enhanced MRI, unlike calcific tendinitis, CCMN presents as an intratendinous mass-like appearance, mild perilesional edema, and diffuse heterogeneous enhancement within the lesion (11).There are also significant differences in treatment between calcific tendinitis (HADD) and calcified chondroid mesenchymal neoplasm (CCMN). Calcific tendinitis (HADD) is typically a self-limiting process, where pain is managed with aspirin, nonsteroidal anti-inflammatory drugs (NSAIDs), or acetaminophen, and it resolves within a few weeks (19).Non-surgical treatments such as ultrasound-guided and CT-guided barbotage, as well as steroid injections, have been shown to relieve pain and restore function (20–24).In contrast, the primary treatment for calcified chondroid mesenchymal neoplasm (CCMN) is surgical resection. Given the significant differences in treatment approaches between CCMN and HADD, it is crucial to distinguish between them.

Beyond HADD, other soft tissue tumors with chondroid or calcified matrix should be included in the differential diagnosis. Extraskeletal chondroma is a benign tumor composed of mature hyaline cartilage, which typically lacks the lobular growth pattern, osteoclast-like giant cells, and primitive chondroid matrix characteristic of CCMN. Soft tissue chondrosarcoma, particularly myxoid or mesenchymal types, can exhibit chondroid matrix and higher cellularity, but is distinguished by its infiltrative growth, cytologic atypia, and the absence of the FN1 rearrangements seen in CCMN. Tenosynovial giant cell tumor (TGCT) with chondroid metaplasia can closely mimic CCMN due to the presence of osteoclast-like giant cells and mononuclear cells. However, the chondroid matrix in TGCT is typically a focal metaplastic change rather than a defining feature, and CCMN lacks the diffuse, strong expression of *CSF1* often seen in TGCT (25). Calcifying fibrous tumor is a paucicellular lesion characterized by hyalinized collagen and psammomatous or dystrophic calcifications, but it lacks the chondroid matrix and giant cells. Finally, phosphaturic mesenchymal tumor (PMT), which can have a myxoid and chondroid matrix with calcification (so-called “grungy” calcification), is a critical differential. PMT is distinguished by its association with tumor-induced osteomalacia, the presence of *FGF23* expression (detectable by IHC or serum levels), and its distinct genetic alterations (e.g., *FN1-FGFR1* fusions, though the specific fusion partners can differ) (26). The clinical presentation of normophosphatemia and the molecular finding of *FN1 (exon 36):FGFR2 (exon 2)* fusion in our case effectively rules out PMT.

Among the patients with available follow-up data, two cases experienced recurrence (8, 14). Therefore, the current primary treatment method, surgical resection, may have limitations. Consequently, based on the structure of these fusion genes, further research into targeted therapy is essential, and targeting receptor tyrosine kinases (TKs) may represent a promising alternative treatment approach (7).

The present case reports presents a CCMN arising at the rare perineal location, with comprehensive radiological, histopathological, immunohistochemical, molecular, and follow-up data. Notably, immunohistochemical analysis demonstrated NKX3.1 positivity in the tumor. However, as this is a single case report, it is not possible to establish whether NKX3.1 has a unique diagnostic role in this tumor entity. Further studies with larger case series are therefore warranted to validate our findings.

## Conclusion

We report a rare case of calcified chondroid mesenchymal neoplasm (CCMN) with an *FN1* (exon 36)::*FGFR2* (exon 2) gene fusion, occurring in the perineal region of a 59-year-old female. The histological features of this tumor are similar to those reported in previous literature. Meanwhile, we reviewed 87 previously reported CCMN cases. These cases showed that CCMN predominantly arises in the soft tissues of the distal extremities or temporomandibular joint region. The mean age of patients is 53 years, and the tumor size ranges from 0.5 to 6.9 cm. CCMN can affect both males and females, with a higher prevalence in females. Contrast-enhanced magnetic resonance imaging (MRI) is of certain value in the diagnosis of CCMN. Histologically, the tumor presents as a solid, lobular lesion with chondroid matrix and varying degrees of calcification. Tumor cells within the matrix exhibit morphologies ranging from oval to spindle-shaped, with some showing eccentric nuclei. Osteoclast-like giant cells are present in most cases. Pathological mitotic figures and necrosis are relatively rare. Immunohistochemically, there are no specific markers for this type of tumor. The most frequently detected molecular genetic abnormality is the fusion of *FN1* with various receptor tyrosine kinase partners (TK), among which *FGFR1/FGFR2* are the most common. In addition, multiple cases of *PDGFRA::USP8* fusion have been identified so far, and this fusion seems to mainly affect large joints. Given that Calcified chondroid mesenchymal neoplasm (CCMN) exhibits well-defined histological and molecular features, adopting ‘CCMN’ as a universal term—replacing the various heterogeneous nomenclatures previously used to describe tumors with similar characteristics—will not only simplify pathological diagnostic workflows but also support the standardized management of these tumors. Furthermore, our case suggests that the tumor may have a broader range of anatomical locations, and clinicians should remain vigilant about this possibility in clinical practice.

## Data Availability

The original contributions presented in the study are included in the article/supplementary material. Further inquiries can be directed to the corresponding author.
